# Visualising single molecules of HIV-1 and miRNA nucleic acids

**DOI:** 10.1186/1471-2121-14-21

**Published:** 2013-04-17

**Authors:** Kate L Jones, Adam Karpala, Bevan Hirst, Kristie Jenkins, Mark Tizard, Cândida F Pereira, Andrew Leis, Paul Monaghan, Alex Hyatt, Johnson Mak

**Affiliations:** 1 Centre for Virology, Burnet Institute, Melbourne, Australia; 2 School of Medicine, Faculty of Health, Deakin University, Waurn Ponds, Geelong, Victoria 3216, Australia; 3 Commonwealth Scientific and Industrial Research Organization, Livestock Industries, Australian Animal Health Laboratory, Geelong, Australia; 4 Monash Micro Imaging, Monash University, Clayton, Australia; 5 Department of Medicine, Monash University, Clayton, Australia; 6 School of Life and Environmental Science, Deakin University, Australia

**Keywords:** HIV, miRNA, RNA, Oligo-fluorescent *in situ* hybridization (O-FISH), Visualization, Viral infection

## Abstract

**Background:**

The scarcity of certain nucleic acid species and the small size of target sequences such as miRNA, impose a significant barrier to subcellular visualization and present a major challenge to cell biologists. Here, we offer a generic and highly sensitive visualization approach (oligo fluorescent *in situ* hybridization, O-FISH) that can be used to detect such nucleic acids using a single-oligonucleotide probe of 19–26 nucleotides in length.

**Results:**

We used O-FISH to visualize miR146a in human and avian cells. Furthermore, we reveal the sensitivity of O-FISH detection by using a HIV-1 model system to show that as little as 1–2 copies of nucleic acids can be detected in a single cell. We were able to discern newly synthesized viral cDNA and, moreover, observed that certain HIV RNA sequences are only transiently available for O-FISH detection.

**Conclusions:**

Taken together, these results suggest that the O-FISH method can potentially be used for *in situ* probing of, as few as, 1–2 copies of nucleic acid and, additionally, to visualize small RNA such as miRNA. We further propose that the O-FISH method could be extended to understand viral function by probing newly transcribed viral intermediates; and discern the localisation of nucleic acids of interest. Additionally, interrogating the conformation and structure of a particular nucleic acid *in situ* might also be possible, based on the accessibility of a target sequence.

## Background

Visualising nucleic acids *in situ* may provide highly significant biological information at a cellular level. Detecting nucleic acid in a single cell routinely employs fluorescence *in situ* hybridization (FISH). Traditionally, FISH requires the use of single probes labelled with multiple fluorophores [1-6] or multiple probes labelled with a single fluorophore [7-9] to allow visualization (for review see [10]). Recent advances in the use of rolling circle amplification from padlock probes [11] and branched DNA probes [12] have significantly improved signal to noise ratios as well as sensitivity during FISH detection. However, the requirement for relatively large target sequences makes these approaches unsuitable for visualizing small size RNAs, such as miRNAs. Alternative approaches include molecular beacons [13], MS2-GFP [14], quantum dots [15] or sub-diffraction microscopy, however, have inherent technical and instrumentation constraints, making them impractical for mainstream use to answer biological questions.

To improve the limitations of nucleic acid detection, we modified a commercially available proximity ligation assay (PLA) to detect individual copies of nucleic acids. PLA was originally designed for detecting co-localization of proteins within a 40 nm distance [16]. The intended detection of co-localized proteins via PLA relies on the use of primary antibodies to the proteins of interest and two species-specific secondary antibodies conjugated to short DNA sequences, which can interact with two short DNA oligonucleotides to form a circularized sequence. This sequence is then ligated, amplified via rolling circle DNA polymerization, and the amplified sequences are hybridized with fluorescent oligonucleotide probes, resulting in an approximate two hundred-fold amplification of the original signal.

**Figure 1:**
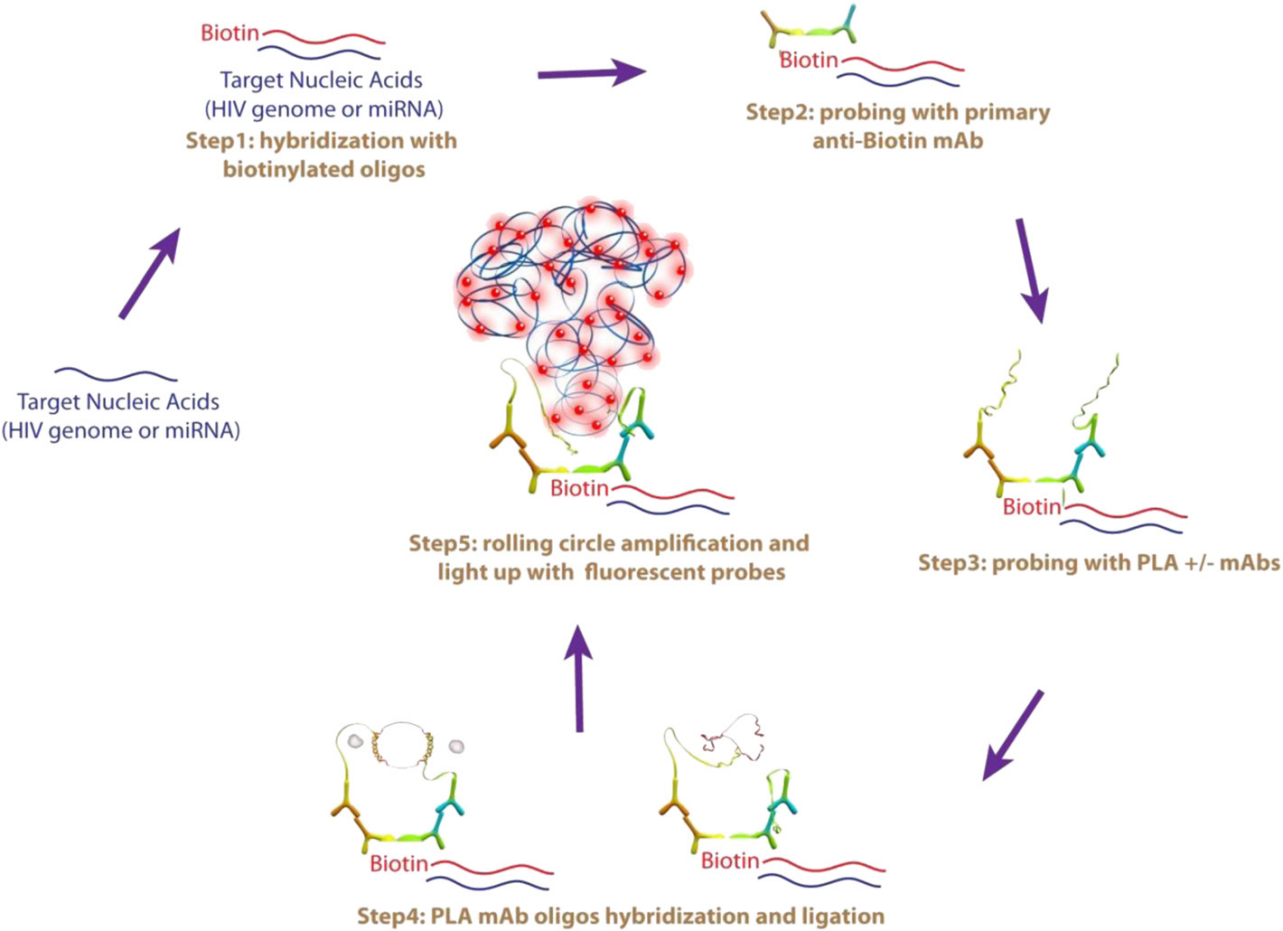
**Overview of the O-FISH mechanism.** Target nucleic acids are initially hybridised with a biotintylated complimentary oligonucleotide probe (step 1). The biotin conjugate is then targeted with an anti-biotin monoclonal antibody (mAb; step 2). The proximal ligation assay (PLA) consisting of a + and – mAb, is then employed to target the specific IgG domain of the biotin-bound mAb (step 3). Oligos conjugated to each of the PLA mAbs are then hybridised to form circularised DNA (step 4) and then rolling circle amplification is used to effectively multiply the target sequence (step 5). Fluorescently labelled oligonucleotide probes are then hybridized with the rolling circle amplified DNA allowing the observation of target with fluorescence microscopy.

Here we have modified the PLA technology to visualise nucleic acids in fixed cells. The method incorporates probing target nucleic acid sequences with a modified FISH protocol combined with detection of probe binding with a commercially available PLA based kit (we have termed this method O-FISH). Initially, target-specific oligonucleotides coupled with biotin are hybridised to the gene of interest. Subsequently an anti-biotin primary antibody is used to bind to the biotin labelled probe, and finally the PLA method detects the conjugated target complex to generate an O-FISH signal (Figure 1). In this study we have used O-FISH to visualize miR146a in both mammalian and avian cells, demonstrating its capacity to detect miRNAs. In addition, we used a HIV-1 model system to illustrate the sensitivity of O-FISH detection, which may reach as little as 1–2 copies of nucleic acids in a single cell. In this model we were able to detect both HIV-1 genomic RNA and newly synthesized viral cDNA allowing visualisation of nucleic acids at various stages of the viral reverse transcription process. Unexpectedly, we also observed that certain HIV RNA sequences are only transiently available for O-FISH detection, implying O-FISH can potentially be used for *in situ* probing of temporal nucleic acid structures.

## Results and discussion

**Figure 2:**
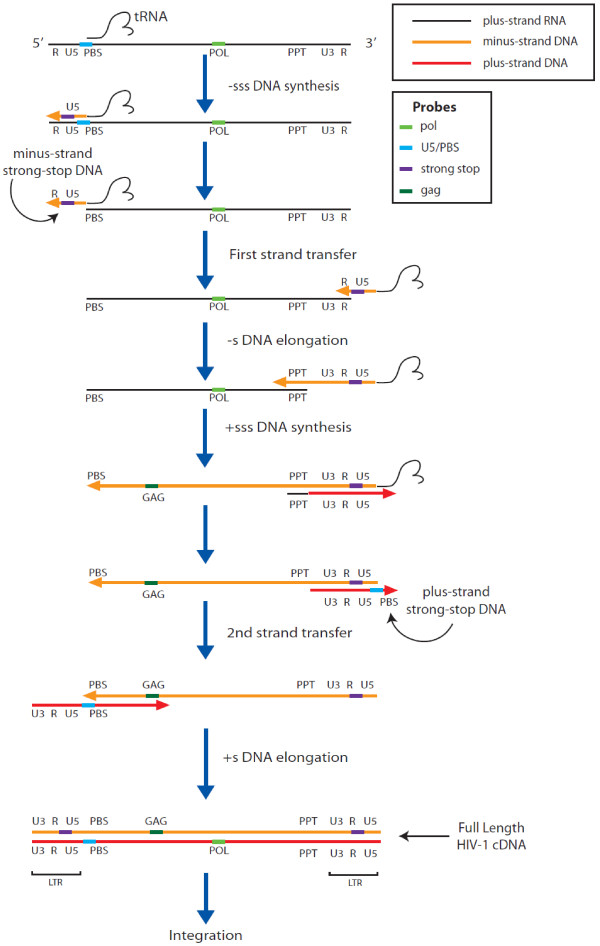
**Probe sequence detection during reverse transcription.** Coloured bars spatially represent the nucleic acid sequence detected by each O-FISH probe. The *pol* probe (light green) detects positive sense viral genomic RNA until its degradation during minus strand DNA elongation and it also detects very late stage plus strand DNA synthesis (plus strand DNA elongation). The *U5/PBS* probe (light blue) detects the positive sense 5’ LTR section of the viral RNA genome and it also detects the plus strand strong stop (+sss) DNA that occurs immediately before the second strand transfer. The *strong stop* probe (purple) detects minus strand strong stop DNA, which is the first segment of DNA produced during reverse transcription and therefore detects all cDNA from early reverse transcription onwards. The *gag* probe (dark green) detects all cDNA from an intermediate point in reverse transcription (after minus strand DNA elongation) onwards. Figure adapted from Telesnitsky & Goff ^29^.

**Figure 3:**
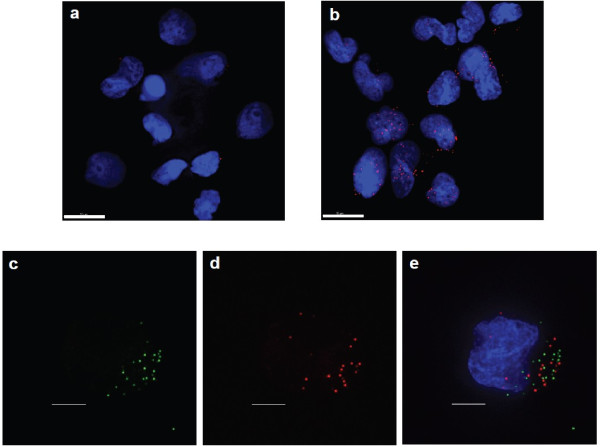
**O-FISH detection of HIV-1 RNA genome.** For HIV-1 nucleic acid detection, MT-2 cells were fixed 6 hrs post mock-infection **(a)**, or infection with HIV-1 **(b)**, onto glass slides and incubated with O-FISH *pol* probe targeted towards HIV-1 positive sense genomic RNA. Bound probe was detected using the O-FISH protocol. O-FISH signals are shown in red and nuclei in blue. Images were derived from a volume compression of a z-stack of 16 images taken at a 0.4 μm step size. Scale bars, 10 μm **(a, b).** MT-2 cells were infected with a mixture of equal amounts of HIV^GFP-Vpr^ and HIV^mCh-Vpr^ (negative co-localization control), fixed after 6 hrs of synchronized infection and imaged **(c, d, e).** HIV^GFP-Vpr^ is shown in green **(c, e)** and HIV^mCh-Vpr^ is shown in red **(d, e)** and a merged HIV^GFP-Vpr^ and HIV^mCh-Vpr^ image is shown **(e)**. The provided image was derived from a volume compression of a z-stack of 25 images taken at a 0.5 μm step size. Scale bars, 5 μm **(c, d and e)**. Nuclei were labelled with Hoechst 33258 (blue). All micrographs are representative of at least 5 images per condition.

To test the O-FISH technology we used a HIV-1 virus system since the viral genome has a well-defined copy number within each HIV-1 virion. For retroviruses, such as HIV-1, two near identical copies of the RNA genome are packaged into the virion [17], which makes HIV-1-infected cells excellent reference samples to assess the sensitivity and specificity of this novel nucleic acid detection system. A biotinylated 26-nucleotide probe targeting the HIV-1 *pol* region (*Pol,* see Figure 2 for relative genome position) was used to detect the presence of HIV-1 RNA genomes in HIV-1 infected lymphoid cells. O-FISH signals were detected in the HIV-1 infected cells (Figure 3b), at levels clearly discernible from minimal background signals observed in the mock-infected control (Figure 3a). The data demonstrates that O-FISH *Pol RNA* detection is highly specific and is able to distinguish HIV-1 nucleic acids from host cellular RNA. In addition we have shown that fluorescently labelled HIV-1 virions do not co-localise during infection, (Figure 3c-e), therefore the observed O-FISH signals are derived from independent viral RNA genomes (Figure 3b). These data indicate that the O-FISH procedure may detect as few as 2 copies of nucleic acid.

**Figure 4:**
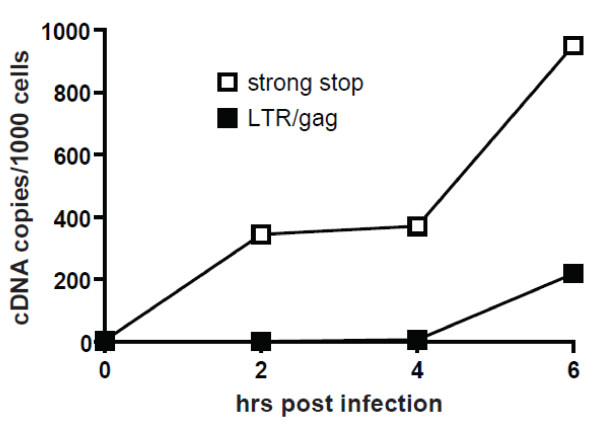
**Quantitative PCR (qPCR) analyses of viral replicative cDNA during early HIV-1 infection.** MT2 cells were either mock-infected or infected with HIV-1 and then lysed for qPCR analyses at 0, 2, 4 or 6 hrs post-infection. HIV-1 cDNA synthesis was quantified by qPCR using primers to detect early (*strong stop*) or intermediate (*gag*) reverse-transcription products. HIV-1 cDNA copies were normalized to cell numbers using CCR5 DNA.

**Figure 5:**
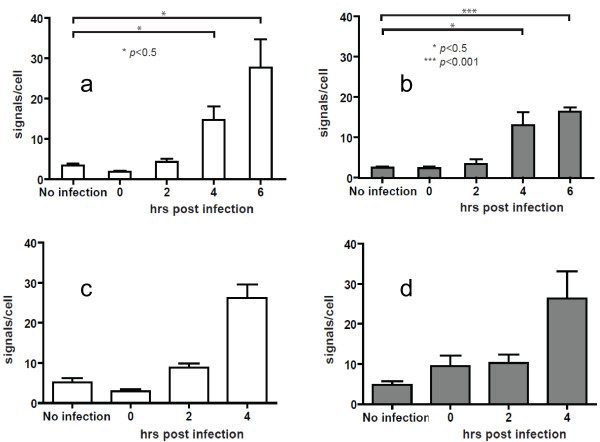
**O-FISH analyses of viral replicative cDNA during early HIV-1 infection.** MT-2 **(a, b)** and Jurkat **(c, d)** cells were either mock-infected or infected with HIV-1 and then fixed onto glass slides at 0, 2, 4 or 6 hrs post-infection. Cells were incubated with O-FISH probes to detect either early (*strong stop*) **(a, c)** or intermediate (*gag*) **(b, d)** HIV-1 cDNA reverse-transcription products (see Figure 2 for relative genome position). Bound probes were detected using the O-FISH method. Quantification of signal events was performed using the spots function in Bitplane Imaris Software. Data shown are mean (+/− SEM) signals per cell, derived from a minimum of 5 images per condition. O-FISH were counted in approx 120–150 cells over 24–36 fields for each of the 4 panels. Paired two-tailed t-tests were performed to determine the significance level of the data sets.

**Figure 6:**
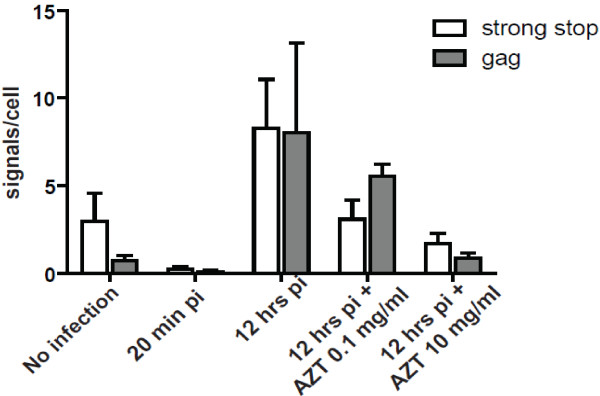
**O-FISH analyses of viral replicative cDNA after blocking with AZT reverse transcriptase inhibitor.** MT2 cells were infected with HIV-1 in the absence or presence of AZT at 0.1 μg/ml or 10 μg/ml concentration. Cells were fixed onto glass slides at either 20 min or 12 h post-infection and subjected to the O-FISH protocol using probes to detect both *strong stop* cDNA and *gag* cDNA. The O-FISH signals were compared to the respective AZT-untreated controls at 12 hrs post-infection. Images were analysed using Imaris software. Data shown are mean (+/− SEM) signals per cell, derived from a minimum of 5 images per condition. O-FISH signals were counted in a total of 158 cells (91 cells over 29 fields for the *gag* probe and 67 cells over 29 fields for the *strong stop* probe).

In order to probe for cDNA viral replicative products in HIV-1 infected cells we used two additional biotinylated 26-nucleotide probes. Early viral cDNA products were detected using a negative sense probe targeting *strong stop cDNA* and intermediate viral cDNA products using a probe targeting *gag cDNA* (see Figure 2 for relative genome position). The time course experiments show the detection of increasing viral cDNA products over time by quantitative PCR (qPCR) (Figure 4). When O-FISH was targeted to *strong stop cDNA* and *gag cDNA* in MT2 cells there was an increase in O-FISH signals at 4–6 hours post-infection relative to the mock-infected control (Figure 5a, b). The O-FISH signals detected using the *strong stop* and *gag* probes throughout the infection time course are consistent with the observations of *strong stop cDNA* and *gag cDNA* identified by qPCR (Figure 4), suggesting that the *strong stop cDNA* and *gag cDNA* detection by the O-FISH method reflects the biology of the HIV-1 replication-cycle. Furthermore, as it is generally accepted that only one copy of viral cDNA is derived from the reverse transcription complex, these data suggest that our O-FISH probes may detect as little as one copy of viral nucleic acid, although, this is somewhat complicated by background levels. To ensure the reproducibility of these observations, a parallel HIV-1 infection was carried out with an alternative lymphoid cell line, Jurkat cells, resulting in similar *strong stop cDNA* and *gag cDNA* O-FISH signals (Figure 5c, d). These data further support the reliability and reproducibility of our O-FISH method. To further confirm the reliability of the O-FISH method we designed experiments to test the specificity of the O-FISH protocol. Due to the small size of the target nucleic acid sequences used for detection, the addition of RNase/DNase enzymatic treatments for background reduction proved impractical due to the difficulty in achieving the complete nucleic acid digestion required to abrogate detection. As an alternative, a reverse transcription inhibitor AZT was used to block synthesis of HIV-1 cDNA and evaluate the specificity of our *strong stop cDNA* and *gag cDNA* O-FISH signal. Samples were treated with AZT then analysed at 12 hours post-infection, to allow maximal HIV-1 replication and thus O-FISH signals. As expected, there was a dose-dependent reduction of detected HIV-1 viral cDNA in the presence of AZT (Figure 6), which additionally demonstrates the specificity of the *strong stop cDNA* and *gag cDNA* O-FISH method.

**Figure 7:**
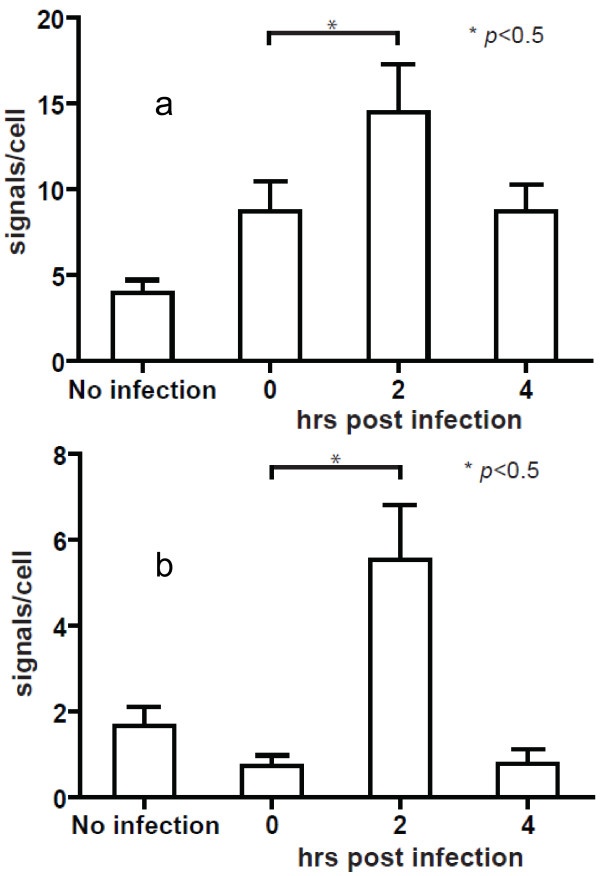
**O-FISH analyses of RNA conformational rearrangements during early HIV-1 infection.** MT2 cells **(a)** and Jurkat cells **(b)** were either mock-infected or infected with HIV-1 and then fixed onto glass slides at 0, 2 or 4 hrs post-infection and incubated with a HIV-1 O-FISH probe to detect plus strand *U5/PBS* RNA(see Figure 2 for relative genome position). Images were analysed using Imaris software. Data shown are mean (+/− SEM) signals per cell, derived from a minimum of 5 images per condition. O-FISH signals were counted in a total of 160 cells (61 cells over 29 fields for the MT2 cells and 91 cells over 20 fields for the Jurkat cells).

The O-FISH method was also used to examine structural rearrangements of nucleic acid targets. We again used HIV-1 as a model since HIV-1 RNA may undergo structural rearrangements in the early steps of the replication cycle [18-20] and thus may limit the O-FISH probe binding to its target due to the initial occupancy of the primer binding site by tRNA and the absence of newly transcribed viral cDNA. Moreover, there is a lack of direct evidence examining the structural rearrangement hypothesis from HIV-1 infected cells [21] thus prompting the use of OFISH to examine structural conformational changes. To examine the HIV-1 structural conformations, a biotinylated 24 nucleotide probe was targeted towards the 5’UTR region of the HIV-1 RNA genome to assess the accessibility of HIV-1 5’ U5/primer binding site (PBS) sequence during the early phase of infection (U5/PBS, Figure 2 for the relative genome position). We have found that the 5’ R-PBS sequence was transiently available for our 5’ U5/PBS O-FISH detection at 2 hours post-infection with reduced levels of detection at 0 and 4 hours post-infection as seen in both MT2 (Figure 7a) and Jurkat (Figure 7b) HIV-1 infected cells. Whilst the reduction of 5’ U5/PBS sequences between 2 and 4 hours post-infection is as expected and can be explained by RNaseH mediated degradation of HIV-1 RNA genome, the increase in detected 5’ U5/PBS sequences between 0 and 2 hours post-infection was an unexpected observation.

As the HIV-1 RNA genome is not replicated in infected cells until later time-points this increase is not due to an increase in the number of RNA genomes in the cell. Instead, we suggest that the increase in detected signals results from either the differential occupancy of primer tRNA on the PBS and/or structural rearrangements of viral RNA genomes during the early steps of HIV infection. RNA rearrangement is likely to be a critical regulator of HIV-1 biology [22] and is therefore an area of much research. The O-FISH method has the potential to provide a means to interrogate the conformational rearrangement of HIV RNA during replication or RNA structural rearrangements in general and thus may provide supporting data for speculated RNA structures.

**Figure 8:**
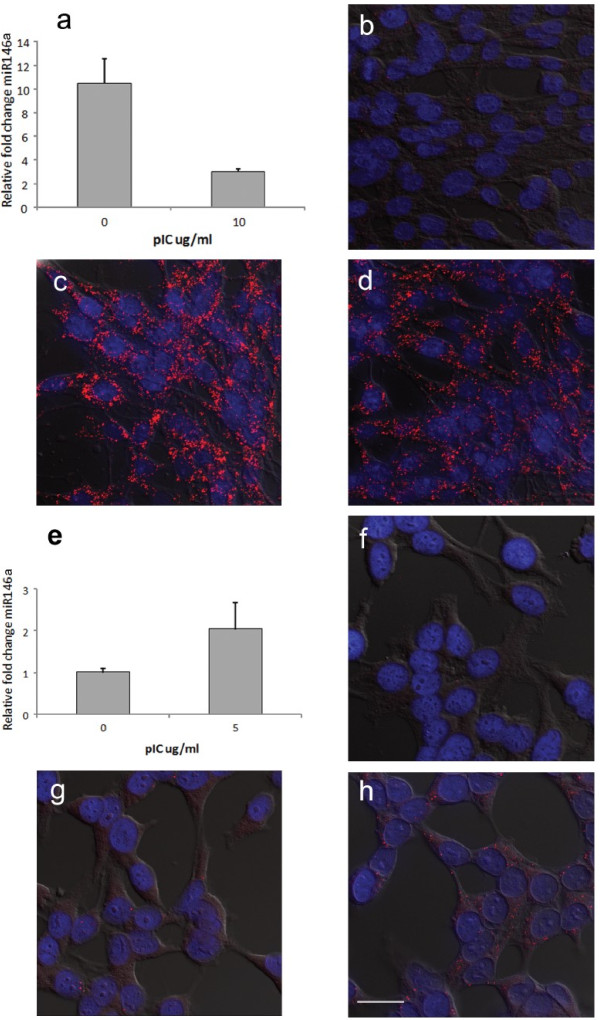
**O-FISH detection of miR146a.** DF1 chicken fibroblasts and HeLa cells were either mock-transfected (**b**, **c**, **f**, **g**) or transfected with pIC (DF1: 10 μg/ml [d], HeLa: 5 μg/ml [h]) for 3 hrs and then analysed by qRT-PCR. qRT-PCR data are shown for DF1 cells **(a)** and HeLa cells **(e)**. O-FISH detection procedure (lacking O-FISH miR146 probe) was carried out as a negative control (DF1 [b], HeLa [f]). The O-FISH signals following mock-transfection in DF1 cells **(c)** and HeLa cells **(g)** are shown, and pIC-treatment of DF1 cells (10 μg/ml) **(d)** and HeLa cells (5 μg/ml) **(h)** are shown. Bound probe was detected using the O-FISH method. Micrographs are representative of at least 5 images per condition. Nuclei were labelled with Hoechst 33258. O-FISH signals are shown in red and nuclei in blue. The provided images were derived from a volume compression of a z-stack of 16 images taken at a 0.4 μm step size. Scale bar of 25 μm applies to DF1 and HeLa cells **(b-d, f-h)**.

To validate whether O-FISH can detect small size RNAs that are as small as miRNA, we used a 19-nucleotide O-FISH probe to detect miR146a expression levels in both chicken DF1 and human HeLa cells. MiR146a levels are linked to the control of type 1 interferon [23], and are altered upon stimulation during virus infection [24] or virus mimics such as dsRNA polyinosinic: polycytidylic (pIC) [23]. Upon pIC treatment, significant differential patterns of miR146a distribution were seen in both DF1 (Figure 8c, d) and HeLa (Figure 8g, h) cells. The contrasting changes in miR146a levels induced by pIC treatment in the cell types tested (down-regulated in DF1 [Figure 8d] and up-regulated in HeLa [Figure 8h]) were in agreement with qRT-PCR analysis (Figure 8a, e). Minimal background signal was detected when the biotinylated miR146a probe was not used in the O-FISH reaction (Figure 8b, f), illustrating O-FISH can be used to detect small RNA sequences that are ~20 nucleotides in length, such as miR146a.

The data shown here not only provide a proof of concept for the O-FISH protocol but also demonstrates specific detection of both the HIV-1 positive sense RNA genome of infecting virions and viral cDNA generated from reverse transcription in the natural target cells of HIV-1. The use of very short oligo probes combined with a novel signal amplification method provide the flexibility for this method to be used to detect very short nucleic acid target sequences in cells. Here we have established that this allows both the detection of specific cDNA products, which are often both short and scarce in nature, during the HIV-1 reverse transcription process and native cellular miRNAs. In addition, we have observed data that suggests this method can also be applied to probing RNA structure and binding events. This method has the potential to be expanded to detection of viral nucleic acids in additional virus models providing an important tool for biologists to unravel complex viral transcriptional processes. Furthermore, the ability of this method to detect short cellular nucleic acid sequences, such as miRNAs, shows that O-FISH can be easily adapted for use outside the field of virology, and may prove to be a useful tool for examining more general processes in cell biology.

## Conclusions

In summary, our data show that O-FISH can detect low copy numbers of nucleic acids that are as little as 20-nucleotides in length. Additionally, O-FISH provides a new method to identify the subcellular distribution of nucleic acids and miRNAs during biological processes. Furthermore, by taking advantage of certain newly synthesized viral nucleic acids that are unique to infectious viral particles, O-FISH could also be used to discern and track the low percentage of functional viruses during infection. Moreover, O-FISH may provide data to support hypothesised RNA structural rearrangements as we have observed in the context of HIV-1.

## Methods

### Cell culture

293T cells were maintained in Dulbecco's modified Eagle medium/high modified (with 4500 mg/l dextrose and 4 mM L-glutamine) medium (DMEM; Invitrogen, Carlsbad, CA, USA) supplemented with 10% (vol/vol) heat-inactivated cosmic calf serum (CCS; Hyclone, Tauranga, New Zealand) and 100 U/ml of penicillin/streptomycin (P/S) (Invitrogen). MT-2 and Jurkat cells (obtained through the AIDS Research and Reference Reagent Program, Division of AIDS, NIAID, NIH) were cultured in Rosewell Park Memorial Institute (RPMI) 1640 medium (Invitrogen) supplemented with 10% vol/vol heat-inactivated fetal calf serum (FCS; Invitrogen) and P/S. HeLa cells (American Type Culture Collection [ATCC], CCL-2) and chicken fibroblast cell line DF1 (ATCC, CRL-12203) were maintained in DMEM supplemented with 10% FCS, 2 mM glutamine, 1.5% sodium bicarbonate and P/S.

### Virus production and purification

HIV virus stocks were produced by poly(ethylenimine) (PEI; Polysciences Inc., Warrington, PA, USA) co-transfection of 293T cells with the full length HIV-1 plasmid DNA pNL4.3 (obtained through the National Institutes of Health AIDS Reagents Program from Dr. Malcolm Martin [25]) and either the EGFP-Vpr fusion protein expressing plasmid pEGFP-Vpr or the mCherry-Vpr fusion protein expressing plasmid mCherry-Vpr (a kind gift from Prof. Tom Hope, Northwestern University, Chicago) to produce HIV^GFP-Vpr^ and HIV^mCh-Vpr.^. Cells were counted and plated at 1 × 10^6^ cells per plate onto 10 cm tissue-culture plates in 6 ml of media. Twenty-four hours later cells were transfected with 2.5 μg of plasmid DNA (1.875 μg of pNL4.3 and 0.625 μg of pEGFP-Vpr) at a 9:1 PEI:DNA ratio. Twelve hours post-transfection cells were washed twice with phosphate buffered saline (calcium and magnesium free; PBS-) and fresh media added. Supernatants were collected 36 hours post-transfection and cellular debris removed by sequential filtration through 0.8 μm and 0.45 μm sterile syringe filters (Sartorius, Goettingen, Germany) . Virus particles were then concentrated by ultracentrifugation through a 20% sucrose cushion using an L-90 ultracentrifuge (SW-41 rotor, Beckman Coulter, Fullerton, CA, USA) at 100,000 × *g* for 1 h at 4°C. Pellets were then resuspended in Benzonase buffer [20 mM Tris–HCl pH 8.0, 2 mM MgCl_2_, 20 mM NaCl] and treated with 90 units/ml Benzonase (Sigma-Aldrich, St. Louis, MO, USA) for 30 min at 37°C to remove any contaminating plasmid DNA. The concentrated viral stocks were quantified using the Vironostika HIV-1 antigen (p24 CA) MicroELISA assay (bioMèrieux, Marcy l'Etoile, France) according to the manufacturer’s instructions and frozen in single-use aliquots at −80°C.

### Infection of lymphoid cells

Synchronized infections were performed as described previously [26]. MT-2 or Jurkat cells were spinoculated with 300 ng p24 CA, as determined by Vironostika HIV-1 antigen (p24 CA) MicroELISA assay (bioMèrieux, Marcy l'Etoile, France) virus per 1 × 10^6^ cells for 2 h at 1,200 × *g* in either 12 or 24 well plates at a non fusion-permissive temperature (15°C). After spinoculation, cells were washed twice with PBS- to remove unbound virus and incubated with warm media at 37°C, 5% CO_2_ to initiate infection.

### Removal of extracellular plasma membrane proteins and un-entered virus using pronase

As part of the experiment using the RT inhibitor AZT pronase treatment was used 20 minutes post-infection to remove all proteins from the outside of the cells, thus removing any bound but un-entered virions from the cell surface. Twenty minutes post-spinoculation cells were washed and resuspended in ice-cold Hank's Balanced Salt Solution (HBSS, Invitrogen) containing 2 mg/ml of protease from *Streptomyces griseus* (pronase E; Sigma-Aldrich) for 10 min on ice. Cells were then washed extensively with HBSS containing 10% FCS then fresh media added and the cells incubated at 37°C.

### Reverse-transcription inhibition using Zidovudine (AZT)

The RT inhibitor Zidovudine (AZT) was obtained through the AIDS Research and Reference Reagent Program, Division of AIDS, NIAID, NIH. AZT was added to cell culture supernatants at the point of infection at either 0.1 or 10 μg/ml as separate conditions. AZT was maintained in the cell culture supernatant throughout the infection.

### Modulation of miRNA levels via synthetic nucleic acids

Introduction of synthetic nucleic acids to DF1 or HeLa cells were carried out using the transfection reagent Lipofectamine 2000 (Invitrogen) according to the manufacturer’s instructions. Briefly, polyinosinic-polycytidylic acid (pIC) was diluted to 10 times the intended final concentration in media, and Lipofectamine was diluted into media at 1 μL / cm^2^ of well area. After 5 min the Lipofectamine and pIC were combined 1:1, incubated for 15 min and then added to cell culture as per manufacturer’s instructions (Invitrogen).

### Quantitative PCR for HIV-1 reverse transcription products

Quantification of HIV-1 reverse transcription products and standardization of cell numbers was performed using quantitative PCR (qPCR). Cells were harvested at various time points post-infection and lysed in PCR lysis buffer containing 10 mM Tris [pH 8.0], 50 mM KCl with 0.5% vol/vol Triton-X100, 0.5% vol/vol NP-40 and 75 mg/ml proteinase K (Roche, Basel, Switzerland). Samples were incubated at 56°C for 2 h before the proteinase K was inactivated by heating to 95°C for 10 min. Samples were then stored at −20°C. Quantitative PCR was performed on an MX3000P QPCR machine (Agilent, Santa Clara, CA, USA). Each PCR reaction contained 1× Brilliant II SYBR Green Master mix (Agilent), 400 nM each primer and 5 μl of cell lysates (1:10 dilution) in a 15 μl reaction volume. The HIV-1 specific primers M667 (sense; 5^′^-GGCTAACTAGGGAACCCACTG-3^′^) and AA55 (antisense; 5^′^-CTGCTAGAGATTTTCCACACTGAC-3^′^) were used to detect early HIV-1 cDNA ([−]strong-stop DNA). The HIV-1 specific primers M667 (sense; 5^′^-GGCTAACTAGGGAACCCACTG-3^′^) and M661 (antisense; 5^′^-CCTGCGTCGAGAGATCTCCTCTGG-3^′^) were used to detect intermediate HIV-1 reverse transcription products (LTR/gag, post 2nd strand transfer). HIV-1 PCR conditions were an initial denaturation at 95°C for 15 min followed by 40 rounds of cycling at 95°C for 10 s, then 60°C for 30 s. Cell numbers were standardized for the human CCR5 gene using the primers LK46 (sense; 5^′^-GCTGTGTTTGCGTCTCTCCCAGGA-3^′^) and LK47 (antisense; 5^′^-CTCACAGCCCTGTGCCTCTTCTTC-3^′^). CCR5 PCR conditions were an initial denaturation at 95°C for 15 min followed by 40 rounds of cycling at 95°C for 20 s, 58°C for 40 s and 72°C for 40 s.

### Quantitative reverse transcription-PCR (qRT-PCR) analyses of miRNA

RNA was harvested using Tri-reagent (Sigma-Aldrich) according to the manufacturer’s instructions. One μg of extracted RNA was subjected to DNase treatment using a DNase 1 kit (Sigma-Aldrich) according to the manufacturer’s instructions. The DNase treated RNA was then polyadenylated with 300 units of polyadenylase polymerase to a final volume of 20 μl and incubated at 37°C for 30 min, then 95°C for 5 min similar to the method by Shi *et al.* [27]. The miRNA was reverse-transcribed to complimentary DNA (cDNA) using a Superscript III first strand synthesis kit (Invitrogen) according to the manufacturer’s instructions then diluted to 1:20. qRT-PCR was carried out using Sybr Green (applied biosystems) and the comparative threshold cycle method, to derive fold change, previously described by Bannister *et al*., (2011). The forward primer sequence miR146 (5’GCG TGA GAA CTG AAT TCC ATG GG) and the endogenous control miR5.8S (5’ TGG GAA TAC CGG GTG CTG T) were individually amplified with a universal reverse primer (5’ GAG GCG AGC ACA GAA TTA ATA CGA C) to generate Ct values for analyses similar to Bannister *et al.* [28].

### O-FISH detection of HIV-1 nucleic acids and miR146

Cells were fixed with either 4% formaldehyde in PBS- at 4°C overnight (HIV analyses) or in 300 μl methanol for 20 min at 20°C (miR146 analyses) and then washed twice in PBS-. Fixed cells were mounted onto slides using a Cytospin II machine (Shandon, Astmoore, UK) at 67 × *g* for 5 minutes. The slides were removed and air-dried overnight in the dark. Slides were either processed immediately or stored in sealed bags at −20°C until needed. All reactions were performed as open-droplet reactions with a droplet volume of 15 μl. Cells were rehydrated in PBS- for 5 minutes and permeabilised with 0.2% Triton for 6 minutes, then washed twice with PBS- before being treated with 0.005% pepsin (Sigma-Aldrich) in 0.01N HCl for 1 min(pepsin treatment was omitted in the miR146 analyses) and then again washed twice with PBS-. O-FISH probe stocks were then diluted to a final concentration of 500 nM in hybridisation buffer (10 mM TRIS [pH7.4], 600 mM NaCl, 1 mM EDTA, 10 nM DTT, 0.1% SDS, 50% formamide) and added to the cells, which were incubated in a humidity chamber at 37°C for 1 hour. Cells were washed twice with PBS- and blocked in blocking buffer (Olink Bioscience, Uppsala, Sweden) at 37°C for 30 minutes. Probe binding was detected via incubation with an anti-biotin primary antibody (Sigma-Aldrich), diluted 1:500 for HIV-1 nucleic acids or 1:1500 for miR146 in antibody diluent (Olink Bioscience) in a humidity chamber at room temperature for 30 minutes. Detection of primary antibody binding was carried out using Duolink II anti-mouse PLA probes and detection kit (Olink bioscience) as per the manufacturer’s instructions. After O-FISH detection, the cells were counterstained with Hoechst 33258 (Invitrogen) and then mounted in Fluoromount-G (Electron Microscopy Sciences, Hatfield, PA, USA) or mounted directly using fluromount with DAPI (Olink Bioscience) for miRNA microscopy.

### O-FISH Probes used for RNA and cDNA detection

Probes were designed complementary to different parts of the HIV-1 RNA genome, to HIV-1 cDNA synthesised at various stages of the reverse-transcription process, or to miR146. Negative sense probes *pol* (5’ CTG TCA GTT ACA TAT CCT GCT TTT CC 3’) and *U5/PBS* (5’ CGG GCG CCA CTG CTA GAG ATT TTC 3’) were used to detect positive sense HIV-1 RNA. Positive sense probes *gag* (5’ ATG GGT GCG AGA GCG TCG GTA TTA AG 3’) and *strong stop* (5’ TGT GAC TCT GGT AAC TAG AGA TCC CT 3’) were used to detect negative sense HIV-1 cDNA. The miRNA probe (5’ CCC ATG GAA TTC AGT TCT C) was used to detect miR146a. All probes had a biotin molecule conjugated to their 5’ end.

### Image acquisition and analysis

For HIV O-FISH analysis, images were acquired using either a DeltaVision-RT (Applied Precision, Issaquah, WA, USA) or Zeiss Axio Observer Z1 (Zeiss) microscope. Images taken on the DeltaVision-RT were acquired in a z-series on a charge-coupled device (CCD) camera (CoolSnap HQ; Photometrics, Tucson, AZ, USA) through either a 60X 1.42 numerical aperture (NA) or a 100X 1.4 NA oil immersion lens. Reference brightfield images were also acquired. Images were deconvolved using softWoRx deconvolution software (Applied Precision) before analysis. All images taken on the Zeiss Axio Observer Z1 were taken in a z-series on a charge-coupled device (CCD) camera (AxioCam MRm Rev. 3, Carl Zeiss, Germany) through a 100X 1.30 NA oil immersion lens. Reference differential interference contrast images (DIC) were also acquired. Images of a minimum of 5 fields were taken per slide for analysis for all experiments. Cell images were analysed by quantification of fluorescent signal using Bitplane Imaris software (Bitplane AG, Zurich, Switzerland). For miRNA analyses, similar procedures were used but the samples were imaged using a Leica (Leica Microsystems, Sydney) SP5 confocal microscope. Fluorescence and DIC images were collected and all images were taken with the same microscope parameters. Quantification of O-FISH signal events was performed using the spots function in Imaris (Bitplane AG, Switzerland). O-FISH signals were designated to be at least 0.5 μm in diameter and intensity was determined on an experimental basis for each set of slides. The total number of O-FISH signals was divided by the number of nuclei for each image. The mean for each sample was then calculated from an average of between 13.4 and 30 cells per time point and condition from at least five randomly acquired images (resulting in data being derived from a total of between 120 and 160 cells per panel in each figure) and is reported as ‘signals per cell’.

### Statistical analysis

Statistical analysis was performed using GraphPad Prism software (GraphPad Software Inc., San Diego, CA, USA). Paired two-tailed t-tests were used to compare variance between two sets of observations as indicated.

## Authors’ contributions

KLJ, AK, BH, AL and PM performed experiments; KLJ, MJ, MT, AH and JM designed experiments; KJ, MT, CFP, AL, PM and AH provided critical reagents and tools; KLJ, AK, BH and JM wrote manuscript. All authors read and approved the final manuscript.

## References

[B1] Femino AM Fay FS Fogarty K Singer RH Visualization of single RNA transcripts in situ Science 1998 280 585 590 10.1126/science.280.5363.585 9554849

[B2] Femino AM Fogarty K Lifshitz LM Carrington W Singer RH Visualization of single molecules of mRNA in situ Methods Enzymol 2003 361 245 304 12624916 10.1016/s0076-6879(03)61015-3

[B3] Maamar H Raj A Dubnau D Noise in gene expression determines cell fate in Bacillus subtilis Science 2007 317 526 529 10.1126/science.1140818 17569828 PMC3828679

[B4] Zenklusen D Larson DR Singer RH Single-RNA counting reveals alternative modes of gene expression in yeast Nat Struct Mol Biol 2008 15 1263 1271 10.1038/nsmb.1514 19011635 PMC3154325

[B5] Tan RZ van Oudenaarden A Transcript counting in single cells reveals dynamics of rDNA transcription Mol Syst Biol 2010 6 358 20393578 10.1038/msb.2010.14 PMC2872610

[B6] Raj A Peskin CS Tranchina D Vargas DY Tyagi S Stochastic mRNA synthesis in mammalian cells PLoS Biol 2006 4 e309 10.1371/journal.pbio.0040309 17048983 PMC1563489

[B7] Raj A van den Bogaard P Rifkin SA van Oudenaarden A Tyagi S Imaging individual mRNA molecules using multiple singly labeled probes Nat Methods 2008 5 877 879 10.1038/nmeth.1253 18806792 PMC3126653

[B8] Khalil AM Guttman M Huarte M Garber M Raj A Rivea Morales D Thomas K Presser A Bernstein BE van Oudenaarden A Regev A Lander ES Rinn JL Many human large intergenic noncoding RNAs associate with chromatin-modifying complexes and affect gene expression Proc Natl Acad Sci U S A 2009 106 11667 11672 10.1073/pnas.0904715106 19571010 PMC2704857

[B9] Raj A Rifkin SA Andersen E van Oudenaarden A Variability in gene expression underlies incomplete penetrance Nature 2010 463 913 918 10.1038/nature08781 20164922 PMC2836165

[B10] Itzkovitz S van Oudenaarden A Validating transcripts with probes and imaging technology Nat Methods 2011 8 S12 S19 10.1038/nmeth.1573 21451512 PMC3158979

[B11] Larsson C Grundberg I Soderberg O Nilsson M In situ detection and genotyping of individual mRNA molecules Nat Methods 2010 7 395 397 10.1038/nmeth.1448 20383134

[B12] Player AN Shen LP Kenny D Antao VP Kolberg JA Single-copy gene detection using branched DNA (bDNA) in situ hybridization J Histochem Cytochem 2001 49 603 612 10.1177/002215540104900507 11304798

[B13] Tyagi S Kramer FR Molecular beacons: probes that fluoresce upon hybridization Nat Biotechnol 1996 14 303 308 10.1038/nbt0396-303 9630890

[B14] Bertrand E Chartrand P Schaefer M Shenoy SM Singer RH Long RM Localization of ASH1 mRNA particles in living yeast Mol Cell 1998 2 437 445 10.1016/S1097-2765(00)80143-4 9809065

[B15] Chan P Yuen T Ruf F Gonzalez-Maeso J Sealfon SC Method for multiplex cellular detection of mRNAs using quantum dot fluorescent in situ hybridization Nucleic Acids Res 2005 33 e161 10.1093/nar/gni162 16224100 PMC1258180

[B16] Soderberg O Gullberg M Jarvius M Ridderstrale K Leuchowius KJ Jarvius J Wester K Hydbring P Bahram F Larsson LG Landegren Direct observation of individual endogenous protein complexes in situ by proximity ligation Nat Methods 2006 3 995 1000 10.1038/nmeth947 17072308

[B17] Paillart JC Shehu-Xhilaga M Marquet R Mak J Dimerization of retroviral RNA genomes: an inseparable pair Nat Rev Microbiol 2004 2 461 472 10.1038/nrmicro903 15152202

[B18] Huthoff H Berkhout B Two alternating structures of the HIV-1 leader RNA Rna 2001 7 143 157 10.1017/S1355838201001881 11214176 PMC1370064

[B19] Huthoff H Berkhout B Multiple secondary structure rearrangements during HIV-1 RNA dimerization Biochemistry 2002 41 10439 10445 10.1021/bi025993n 12173930

[B20] Berkhout B Ooms M Beerens N Huthoff H Southern E Verhoef K In vitro evidence that the untranslated leader of the HIV-1 genome as an RNA checkpoint that regulates multiple functions through conformational changes J Biol Chem 2002 277 19967 19975 10.1074/jbc.M200950200 11896057

[B21] Paillart JC Dettenhofer M Yu XF Ehresmann C Ehresmann B Marquet R First snapshots of the HIV-1 RNA structure in infected cells and in virions J Biol Chem 2004 279 48397 48403 10.1074/jbc.M408294200 15355993

[B22] Lu K Heng X Garyu L Monti S Garcia EL Kharytonchyk S Dorjsuren B Kulandaivel G Jones S Hiremath A Divakaruni SS LaCotti C Barton S Tummillo D Hosic A Edme K Albrecht S Telesnitsky A Summers MF NMR detection of structures in the HIV-1 5'-leader RNA that regulate genome packaging Science 2011 334 242 245 10.1126/science.1210460 21998393 PMC3335204

[B23] Hou J Wang P Lin L Liu X Ma F An H Wang Z Cao X MicroRNA-146a feedback inhibits RIG-I-dependent Type I IFN production in macrophages by targeting TRAF6, IRAK1, and IRAK2 J Immunol 2009 183 2150 2158 10.4049/jimmunol.0900707 19596990

[B24] Stanczyk J Pedrioli DM Brentano F Sanchez-Pernaute O Kolling C Gay RE Detmar M Gay S Kyburz D Altered expression of MicroRNA in synovial fibroblasts and synovial tissue in rheumatoid arthritis Arthritis Rheum 2008 58 1001 1009 10.1002/art.23386 18383392

